# Nanoporous Copper Films via Dynamic Hydrogen Bubbling: A Promising SERS Substrate for Sensitive Detection of Methylene Blue

**DOI:** 10.3390/nano15120945

**Published:** 2025-06-18

**Authors:** Noor Tayyaba, Stefano Zago, Andrea Giura, Gianluca Fiore, Luigi Ribotta, Federico Scaglione, Paola Rizzi

**Affiliations:** 1 Dipartimento di Chimica e Centro Interdipartimentale NIS (Nanostructured Surfaces and Interfaces), Università di Torino, Via Pietro Giuria 7, 10125 Torino, Italy; noor.tayyaba@unito.it (N.T.); stefano.zago@unito.it (S.Z.); gianluca.fiore@unito.it (G.F.); paola.rizzi@unito.it (P.R.); 2 Applied Metrology and Engineering Division, Istituto Nazionale di Ricerca Metrologica (INRiM), Strada delle Cacce 91, 10135 Torino, Italy; a.giura@inrim.it (A.G.); l.ribotta@inrim.it (L.R.)

**Keywords:** nanoporous copper film, electrodeposition, dynamic hydrogen bubbling template, CTAB, SERS

## Abstract

Cu-based nanomaterials have received considerable attention as promising and cost-effective substrates for surface-enhanced Raman spectroscopy (SERS) applications despite their relatively low enhancement factor (EF) compared to noble metals like gold and silver. In this study, a fast and affordable synthesis route is proposed to obtain a three-dimensional porous copper film (NPC) via an electrodeposition technique based on the dynamic hydrogen bubbling template (DHBT). Two sets of NPC film were synthesized, one without additives and the other with cetyltrimethylammonium bromide (CTAB). The impacts of deposition time on the NPCs’ porous morphology, thickness, and SERS performance were systematically investigated. With the optimal deposition time, the nanopore sizes could be tailored from 26.8 to 73 μm without additives and from 12.8 to 24 µm in the presence of CTAB. The optimal additive-free NPC film demonstrated excellent SERS performance at 180 s of deposition, while the CTAB-modified film showed strong enhancement at 120 s towards methylene blue (MB), a highly toxic dye, achieving a detection limit of 10^−6^ M. Additionally, the samples with CTAB showed better efficiency than those without CTAB. The calculated EF of NPC was found to be 5.9 × 10^3^ without CTAB and 2.5 × 10^3^ with the CTAB, indicating the potential of NPC as a cost-effective candidate for high-performance SERS substrates. This comprehensive study provides insights into optimizing the structural morphology of the NPCs to maximize their SERS enhancement factor and improve their detection sensitivity toward MB, thus overcoming the limitations associated with conventional copper-based SERS substrates.

## 1. Introduction

Considerable attention has been focused on improving the development/fabrication of 3- dimensional porous metals due to their unique features such as controlled morphology, high surface area, well-organized pore structure, and precise pore-size distributions [1,2,3]. These unique characteristics have applications in catalysis and electro-catalysis [4,5,6], sensors [7,8], SERS [9,10,11], and pharmaceuticals [12,13]. Among the porous metals, there has been a growing interest in porous copper (Cu) and copper alloys due to their exceptional characteristics like high thermal conductivity and catalytic activity [14]. Moreover, the presence of porosities can enhance the copper catalytic efficiency, fluid permeability, wettability, optical [15] and electrocatalytic applications [16].

NPC can be fabricated using different methods, including template synthesis, chemical dealloying, electrodeposition, and surfactant-mediated synthesis [17,18,19,20,21]. Among these, the electrodeposition using the DHBT method is one of the most effective techniques for fabricating NPC, introduced by Shin et al. in 2003 [22]. It is a cost-effective approach that allows precise control over the structure of the deposited material by adjusting various factors such as temperature, electrolyte composition, current/potential, and duration [23,24]. Therefore, it is essential to have precise control of the electrodeposition parameters, to design high-performance porous copper.

The DHBT method involves the evolution of hydrogen gas, which serves as a soft template for reducing the metal ions and forming a 3D hierarchical porous nanostructure. The process begins with the application of an overpotential, which initiates the nucleation of hydrogen gas on the electrode surface [25]. As the potential is applied, hydrogen gas is generated and acts as a cathode aggregate, temporarily adhering to the surface before desorbing into the gas phase. Following this, the hydrogen bubbles undergo growth and coalescence, leading to the formation of a hollow porous framework with a large surface area. This structure is created through focused deposition around short-lived adherent hydrogen bubbles and occurs due to the simultaneous reduction of metal ions in the precursor solution [26]. Ultimately, this dynamic template facilitates the discharge of metallic ions onto the working substrate, enhancing its properties and effectiveness in electrochemical applications [27].

Moreover, it was noticed that the surfactants commonly used as stabilizers are favourable in the DHBT synthesis method to customize and stabilize the hydrogen bubbles produced from the electrochemical reduction of protons [28]. This, in turn, impacts the pore structure of the deposited metal films. Li et al. studied the evolution process of porous copper films and revealed that a higher concentration of CTAB can result in smaller pore size and wall thickness because the hydrogen bubble size is affected by the surfactant concentration [24]. Furthermore, the pore morphology resulting from this technique depends on the stability of the surface-adsorbed hydrogen bubbles. Using DHBT templates for surface nanostructuring allows precise porosity control, pore diameter, and film thickness, resulting in NPC with significant active sites, a notably large surface area, and a high volume of interconnected porosity [29]. The source and concentration of the substrate material and the presence of surfactants and additives can also influence the structural morphology. Adjusting these parameters allows the bubble size to be controlled before it is released from the substrate.

NPC fabricated using the DHBT method has potential for use in SERS due to its large surface area and interconnected porosity [30]. Recent research suggests that nanostructured copper substrates can provide comparable enhancement levels to traditional Ag and Au substrates, leading to increased interest in developing optimized nanostructured copper for efficient SERS applications at a competitive cost [31]. With its unique advantages and cost-effectiveness, NPC is anticipated to serve as a promising substrate for SERS. Chen et al. successfully synthesized nanoporous copper with adjustable pore sizes by controlling the dealloying process, leading to enhanced SERS performance due to the tuneable nanoporosity among the copper nanoparticles [32]. In another study, Tan et al., conducted a study on the SERS properties of Cu butterfly wing scales. The results show that pronounced Raman enhancement mainly originates from the 3D sub-micrometre periodic rib structures located on the main ridges of Cu scales, which exhibit a low detection limit of 10-8 M of rhodamine 6G (R6G) [33].

Herein, the SERS activity of the Cu substrate can be modulated by using the electrodeposition with the DHBT method to design the morphology of the NPC film. This study aims to enhance the effectiveness of the SERS substrate and make it highly sensitive for detecting MB. MB is a synthetic dye that is extensively used in industries such as textiles, paper, and aquaculture [34,35]. Although it serves various functional purposes, due to its toxic, mutagenic, and bio-accumulative properties pose serious risks to human health [36,37,38]. Additionally, its illegal use in aquaculture as an antifungal agent poses significant risks to both aquatic life and human health [39]. Consequently, the persistence of MB in water and its resistance to conventional degradation methods have therefore made its detection in wastewater an increasing environmental priority.

## 2. Material and Methods

### 2.1. Material

The electrodeposition of the NPC was conducted on a metallic substrate, which was prepared by cutting a commercial brass bar with a diameter of 15 mm into a disk of ~1 mm in thickness. The nominal composition of brass is approximately 53–56 atomic percent (at. %) copper and 44–47 atomic percent (at. %) zinc, resulting in an α + β structure. The brass disks were mechanically polished using a Struers machine (model LaboPol-5) and silicon carbide papers (from 600 to 1200 mesh) to achieve a mirror-polished surface. After polishing, the samples were sonicated for 3 min in acetone, MilliQ water, and then finally in ethanol solutions to remove the impurities and dry in the air. The as-prepared disks primarily served as a conductive and cost-effective substrate, providing the necessary support and ensuring the handling stability of the NPCs. The electrolyte for electrodeposition was prepared by mixing 0.5 M sulphuric acid (H_2_SO_4_) and 0.1 M copper sulfate (CuSO_4_) in 200 mL of MilliQ water. In another experiment, an alternative electrolyte solution was prepared using 0.5 mM CTAB, along with the same concentrations of sulfuric acid (0.5 M) and copper sulfate (0.1 M) in 200 mL of MilliQ water.

MB was used as the probe molecule to evaluate the SERS activity of the NPC samples. To prepare the solutions, MB powder was accurately measured and dissolved in 20 mL of Milli-Q to create a concentrated stock solution of 10^−3^ M. Subsequent dilutions were made to achieve concentrations ranging from 10^−4^ M to 10^−7^ M. All the solutions were prepared using chemical-grade reagents from Sigma Aldrich (Darmstadt, Germany).

### 2.2. Fabrication of NPC

The electrochemical depositions were performed using a conventional three-electrode setup connected with an Autolab potentiostat (Mehrohm, Utrecht, The Netherlands). The porous Cu was electrodeposited on a well-polished brass disk (acting as a working electrode) using the 0.1 M CuSO_4_ and 0.5 M H_2_SO_4_ with and without CTAB as electrolytes at the potentiostatic mode by applying −4 V. A platinum foil, 3 cm long and 1 cm wide, was served as a counter electrode and placed at a 2 cm distance in front of the working electrode constituted by the polished disk inserted in a sample holder and exposing a circular area of 11 mm of diameter to complete the circuit with a Ag/AgCl as a reference electrode in a double-bridge configuration loaded with a saturated solution of potassium chloride (KCl). All the samples were electrodeposited at 25 °C by varying the time (30 s, 60 s, 120 s, and 180 s) under unstirred conditions. The samples prepared with the two electrolytes at different deposition times were labeled according to the following nomenclature: NPC_30 s, NPC_60 s, NPC_120 s, and NPC_180 s for the series without surfactant, and NPC_CTAB_30 s, NPC_CTAB_60 s, NPC_CTAB_120 s, and NPC_CTAB_180 s for the series containing the CTAB surfactant. After electrodeposition, the samples were rinsed with distilled water, air-dried, and stored in well-closed sample holders for further analysis. The morphology of all the samples was consistently reproducible across three independent electrodeposition trials conducted under identical conditions, showing consistent porous features in each case.

### 2.3. Characterization of NPC

The characterization of all samples was carried out after the electrodeposition of porous NPCs. The X’pert X-ray Diffraction (XRD) in Bragg–Brentano geometry (Panalytical in Almelo, The Netherlands) with monochromatic Cu Kα radiation was used to investigate the impact of time on the as-deposited porous NPC. To facilitate the acquisition of XRD data, the samples were removed from the substrate disks using a scalpel and subsequently subjected to diffraction analysis. The indexing of diffraction peaks was carried out using X’Pert Highscore software (version 2.2c (2.2.3.)). The lattice parameters and metal/oxide weight fraction of the most promising samples were assessed by performing Rietveld analyses on XRD patterns using the MAUD software (version 2.9993) [40].

Furthermore, the surface morphology of the NPC samples was evaluated using Scanning Electron Microscopy (SEM) (TESCAN in Brno, Czech Republic) coupled with energy-dispersive X-ray spectroscopy (EDS) (Oxford Instruments Oxford Ultim-Max 100 in Abingdon, UK) for compositional analysis. The average pore size was determined using the Image J (1.52v) software.

A focus variation instrument (Bruker Alicona in Graz, Austria) was used to measure the thickness of NPC film with and without CTAB. The topographies were performed by using a 4× objective with a working distance of 30 mm. Further details of the acquisition procedure and data analysis are reported in the Supplementary Materials in Figures S1–S4.

Renishaw in Via Raman Microscope (Renishaw, Wotton-under-Edge, England) with a 514 nm laser line was used for the SERS investigation of carcinogenic MB. The detection of MB was set up with a 20× ULWD objective, 10% power at the sample surface, and accumulation of 3 acquisitions with an acquisition time of 20 s. Each SERS spectrum represents an average of 15 measurements acquired from randomly selected locations on the NPC substrate. The spectrometer was calibrated before measurements using the Raman band of a silicon wafer at 520 cm^−1^.

### 2.4. SERS Measurements

The NPCs, fabricated with and without CTAB using the DHBT method for deposition times of 30, 60, 120, and 180 s, were then immersed in a 5 mL solution of 10^−4^ M MB overnight to ensure maximum absorption of the probe molecules onto the surface. After immersion, the samples were removed from the solution and allowed to air-dry for 10 min. Once dry, the samples were placed in the sample holder of the SERS instrument for measurements. This procedure was consistently applied for each MB concentration of 10^−4^ M, 10^−5^ M, 10^−6^ M, and 10^−7^ M. The SERS enhancement factor (EF) for NPC film fabricated both with and without CTAB was calculated using the following formula:

$$E\cdot F=\frac{I_{SERS}}{C_{SERS}}\times \frac{C_{Cu\;disk}}{I_{Cu\;disk}}$$

In this equation, *I_SERS_* represents the intensity corresponding to the highest peak observed at ~1628 cm^−1^ of the selected scattering band in the SERS spectrum obtained with the NPC film, while *I_Cu disk_* denotes the intensity of the corresponding scattering band in the Raman spectrum using a copper disk substrate as a control. The concentration of the MB used for the SERS measurements, *C_SERS_*, was set at 10^−7^ M. In contrast, the concentration for the control measurements on the copper disk substrate, *C_Cu disk_*, was set at 10^−4^ M.

## 3. Results and Discussions

### 3.1. Morphological Characterization

**Figure 1:**
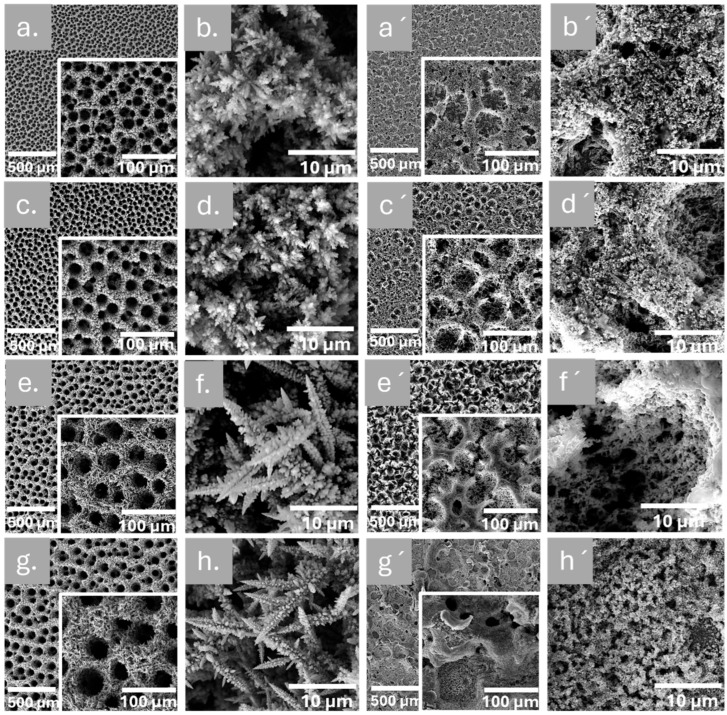
SEM analysis of NPCs deposited with and without CTAB: (**a**) represents the NPC_30 s, (**b**) NPC_30 s at higher magnification, (**c**) NPC_60 s, (**d**) NPC_60 s at higher magnification, (**e**) NPC_120 s, (**f**) NPC_120 s at higher magnification, (**g**) NPC_180 s, and (**h**) NPC_180 s at higher magnification. Similarly to this, (**a’**) NPC_CTAB_30 s, (**b’**) NPC_CTAB_30 s at higher magnification, (**c’**) NPC_CTAB_60 s, (**d’**) NPC_CTAB_60 s at higher magnification, (**e’**) NPC_CTAB_120 s, (**f’**) NPC_CTAB_120 s at higher magnification, (**g’**) NPC_CTAB_180 s, and (**h’**) NPC_CTAB_180 s at higher magnification.

The SEM images of NPC deposited films in Figure 1 provide a detailed depiction of the morphology of as-prepared NPC via the DHBT method in the absence and presence of CTAB additive, prepared by applying −4 V potential for 30 s, 60 s, 120 s, and 180 s. Notably, all the deposited NPCs without CTAB exhibit a relatively homogeneous and uniform distribution of 3-D interconnected porous structures with numerous holes and pore walls, possessing a honeycomb-like morphology. The pore size of NPC deposits is directly influenced by the duration of electrodeposition time, as observed in Figure 1. NPC deposited films in 30 s have smaller pores (Figure 1a,b) compared to those NPC deposited in 60s, which have relatively larger pores size (Figure 1c,d). Additionally, as the deposition time increased from 60 s to 120 s and 180 s, the diameter of the honeycomb-like pores also increased, as clearly illustrated in Figure 1a,c,e,g. This intriguing phenomenon can be attributed to the formation of hydrogen bubbles during the electrochemical reduction of hydrogen ions, which serve as dynamic templates during the copper electrodeposition process, leading to the growth of porous copper films between the coalescing and expanding hydrogen bubbles. The coalescence of the hydrogen bubbles determines the growth of the pore diameter.

Furthermore, higher magnified images show details of a honeycomb-like structure that resembled a blossom flower of small and large dendrites growing in all directions (see Figure 1b,d). As the deposition time increased, the dendrites became more refined and longer with respect to those produced after 30 s and 60 s deposition times. These dendrites had a very long and thin principal branch with multiple sub-branches, giving a leaf-like appearance throughout. The dendrite formation in NPC film typically begins in the plateau region and is observable in the DHBT method without CTAB (Figure 1b,d,f,h). When voltage is applied, Cu^2+^ ions are reduced at the cathode, leading to the production of metallic copper and the subsequent formation of either separated nuclei or a uniform copper layer. The initial growth areas then transform into preferential growth zones as the intensified electric fields at the tips of the dendrites attract more Cu^2+^ ions and enhance diffusion flow, thereby facilitating further elongation of the dendrites [41]. Moreover, the anisotropic characteristics of copper growth promote the directed growth of dendrites by allowing atoms to preferentially attach to the corners and edges of existing crystals. The remarkable 3-D network and hierarchical pore structure of the copper deposits demonstrate the effectiveness of the DHBT method in creating highly porous and interconnected copper structures with tuneable pore sizes.

In the DHBT method, surfactants play a crucial role in reducing the surface tension and maintaining the stability of the bubble system by inhibiting the coalescence and growth of bubbles throughout the process. The resulting bubble template leads to a reduction in pore size and wall thickness, which ultimately affects the structural morphology of the deposited NPC. Nonetheless, the direct influence of surface tension on bubble size is relatively low. The SEM analysis of NPC deposited with 0.5 mM of CTAB, with deposition times of 30 s, 60 s, 120 s, and 180 s, is illustrated in Figure 1a’–h’.

Notably, the deposited NPCs with CTAB resulted in a markedly different morphology than those prepared without CTAB (Figure 1a–h). The NPCs with CTAB show a relatively non-homogeneous distribution of 3-D interconnected porous structures with numerous holes, as shown in Figure 1a’,c’,e’,g’. Furthermore, the pore size of the NPCs prepared with CTAB is relatively smaller than those without CTAB even at the higher deposition time, i.e., 180 s sub micrometric pores were observed. This might be due to the cationic surfactant (CTAB) that prevents bubble coalescence during the deposition process by adsorbing at the interface between the gas and liquid phases, effectively adhering to the surface of the bubbles. This mechanism maintains smaller hydrogen bubbles, resulting in smaller pore sizes with respect to the sample produced without CTAB. Moreover, also in the case of samples produced with CTAB, the deposition time is directly proportional to the pore size, that increases from 12.8 µm to 24.4 µm with an increase in deposition time from 30 s to 120 s respectively. The detailed summary of NPC pore size and dendrite size with and without CTAB was presented in Table 1. The pore size value reported in Table 1 was estimated from high-magnification SEM images using ImageJ software. At least 50 pores or dendrites from different regions of the sample surface were analysed to ensure statistical reliability.

The SEM images of the NPC-deposited films in Figure 1 provide a detailed depiction of the morphology of as-prepared NPC via the DHBT method in the absence and presence of CTAB additive, prepared by applying −4 V potential for 30 s, 60 s, 120 s, and 180 s. Notably, all the deposited NPCs without CTAB exhibit a relatively homogeneous and uniform distribution of 3D interconnected porous structures with numerous holes and pore walls, possessing a honeycomb-like morphology. The pore size of NPC deposits is directly influenced by the duration of electrodeposition time, as observed in Figure 1. NPC-deposited films in 30 s have smaller pores (Figure 1a,b) compared to those NPC-deposited in 60 s, which have relatively larger pore sizes (Figure 1c,d). Additionally, as the deposition time increased from 60 s to 120 s and 180 s, the diameter of the honeycomb-like pores also increased, as clearly illustrated in Figure 1a,c,e,g). This intriguing phenomenon can be attributed to the formation of hydrogen bubbles during the electrochemical reduction of hydrogen ions, which serve as dynamic templates during the copper electrodeposition process, leading to the growth of porous copper films between the coalescing and expanding hydrogen bubbles. The coalescence of the hydrogen bubbles determines the growth of the pore diameter.

Furthermore, higher magnified images show details of a honeycomb-like structure that resembled a blossoming flower of small and large dendrites growing in all directions (see Figure 1b,d). As the deposition time increased, the dendrites became more refined and longer with respect to those produced after 30 s and 60 s deposition times. These dendrites had a very long and thin principal branch with multiple sub-branches, giving a leaf-like appearance throughout. The dendrite formation in the NPC film typically begins in the plateau region and is observable in the DHBT method without CTAB (Figure 1b,d,f,h). When voltage is applied, Cu^2+^ ions are reduced at the cathode, leading to the production of metallic copper and the subsequent formation of either separated nuclei or a uniform copper layer. The initial growth areas then transform into preferential growth zones as the intensified electric fields at the tips of the dendrites attract more Cu^2+^ ions and enhance diffusion flow, thereby facilitating further elongation of the dendrites [37]. Moreover, the anisotropic characteristics of copper growth promote the directed growth of dendrites by allowing atoms to preferentially attach to the corners and edges of existing crystals. The remarkable 3D network and hierarchical pore structure of the copper deposits demonstrate the effectiveness of the DHBT method in creating highly porous and interconnected copper structures with tuneable pore sizes.

In the DHBT method, surfactants play a crucial role in reducing the surface tension and maintaining the stability of the bubble system by inhibiting the coalescence and growth of bubbles throughout the process. The resulting bubble template leads to a reduction in pore size and wall thickness, which ultimately affects the structural morphology of the deposited NPC. Nonetheless, the direct influence of surface tension on bubble size is relatively low. The SEM analysis of NPC deposited with 0.5 mM of CTAB, with deposition times of 30 s, 60 s, 120 s, and 180 s, is illustrated in Figure 1a’–h’.

Notably, the deposited NPCs with CTAB resulted in a markedly different morphology than those prepared without CTAB (Figure 1a–h). The NPCs with CTAB show a relatively non-homogeneous distribution of 3D interconnected porous structures with numerous holes, as shown in Figure 1a’,c’,e’,g’. Furthermore, the pore size of the NPCs prepared with CTAB is relatively smaller than those without CTAB even at the higher deposition time, i.e., 180 s sub-micrometric pores were observed. This might be due to the cationic surfactant (CTAB) that prevents bubble coalescence during the deposition process by adsorbing at the interface between the gas and liquid phases, effectively adhering to the surface of the bubbles. This mechanism maintains smaller hydrogen bubbles, resulting in smaller pore sizes [27] with respect to the sample produced without CTAB. Moreover, also in the case of samples produced with CTAB, the deposition time is directly proportional to the pore size, which increases from 12.8 µm to 24.4 µm with an increase in deposition time from 30 s to 120 s, respectively. The detailed summary of NPC pore size and dendrite size with and without CTAB is presented in Table 1. The pore size value reported in Table 1 was estimated from high-magnification SEM images using the ImageJ software. At least 50 pores or dendrites from different regions of the sample surface were analyzed to ensure statistical reliability.

**Figure 2:**
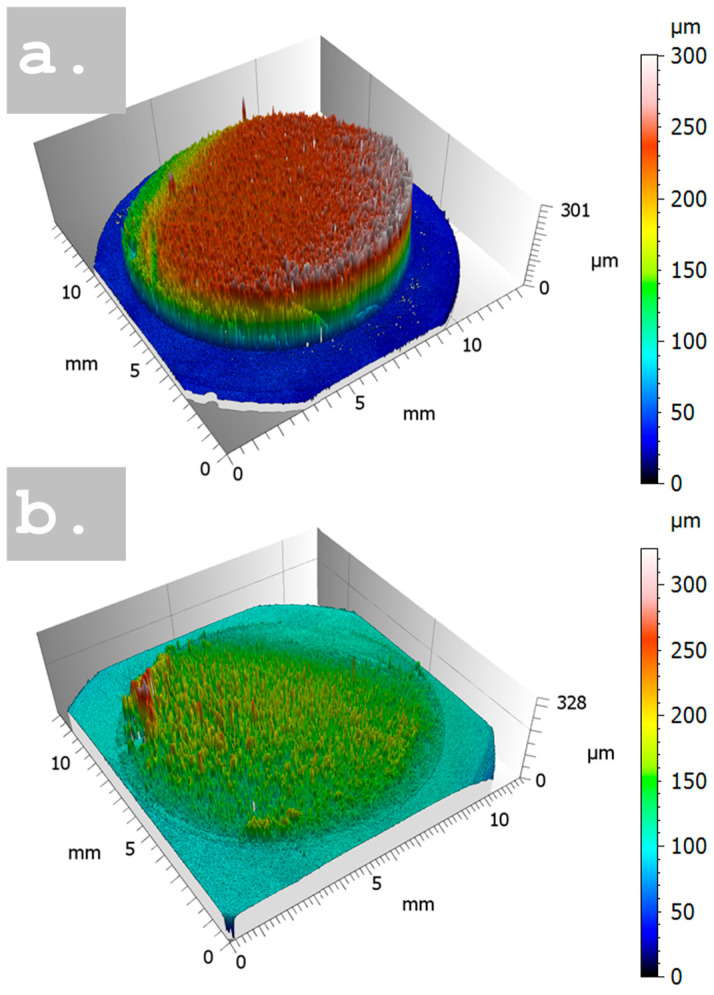
Three-dimensional topographies of samples with and without CTAB: (**a**) represents the NPC_180 s, and (**b**) NPC_CTAB_180 s.

The thickness evolution of the NPC films was studied as a function of deposition time thanks to the extraction of the histogram of heights from the reconstructed topographies processed by using the metrological software Mountains Map v 10.0. In Figure 2a a comparison of the maps for samples NPC_180 s and NPC_CTAB_180 s is performed; all the maps are reported in Figure S5 of the Supplementary Materials. The entire samples were measured by stitching 4 × 4 images, defining a volume of about 14 mm × 14 mm × 0.5 mm in X, Y, and Z directions, for a total of about 21 million points measured.

**Figure 3:**
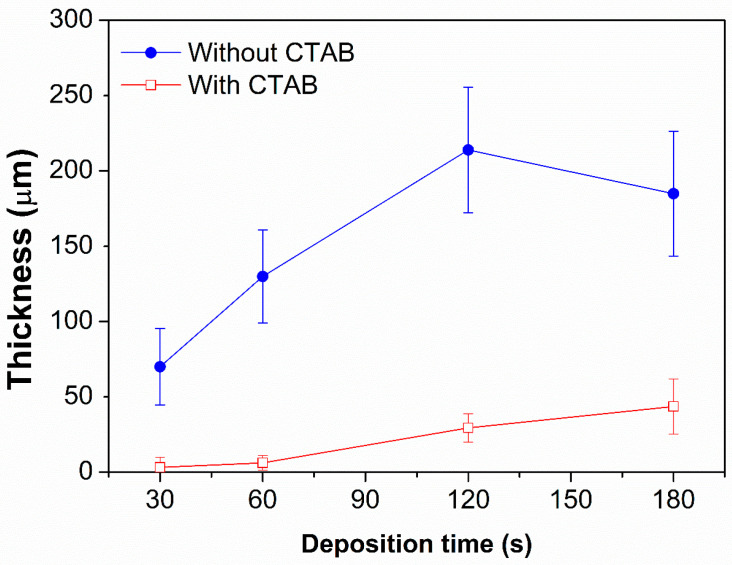
Thickness of NPCs with respect to deposition time; blue line NPCs deposit without CTAB and red line NPCs deposited with CTAB. The lines between points should be intended as a guide for the eyes.

The average thickness of NPCs evaluated from the mapping was plotted within the error bar, which represent the standard deviation of the histogram [42] for the NPCs synthesized with and without CTAB, and reported in Figure 3 and in the Supplementary Materials in Table S1. The thickness of samples without surfactant, which refers to the blue line, shows a linear trend in thickness up to 120 s of deposition. However, beyond this point, specifically at 180 s, a constant or a slight decrease in the average thickness of the deposited film is observed. During prolonged deposition times, localized accumulation of bubbles occurs in certain areas of the sample, which can hinder the homogeneous growth of the deposited layer. The average thickness, along with its standard deviation, reflects this phenomenon. Conversely, NPCs prepared with surfactant (see the red line in Figure 3) show a direct relationship where the thickness of the porous copper films rises alongside the deposition duration. Furthermore, the high values of standard deviation are indicative of the inhomogeneity of the samples, which is more pronounced for the samples without CTAB [43]. The deposition thickness is strongly influenced by the presence of surfactants, even under identical time and potential conditions. For extended deposition times, the thickness of the film obtained without surfactants is approximately four times greater than that of the film deposited with surfactants. This highlights the versatility of the DHBT method, which enables precise control over thin film thickness to meet specific application needs.

### 3.2. Structural Characterization

In order to improve the XRD data acquisition and avoid peak overlap between the brass substrate and the deposited copper, the samples were carefully detached from the disks using a scalpel and then analyzed through X-ray diffraction techniques. Since NPCs produced with short deposition times were non-removable from the substrate due to their excessive thinness, this procedure was limited to samples deposited for longer durations. Specifically, the focus was on NPCs obtained at 180 s without surfactant and at 120 s with surfactant, as these exhibited enhanced SERS performance, as detailed below.

**Figure 4:**
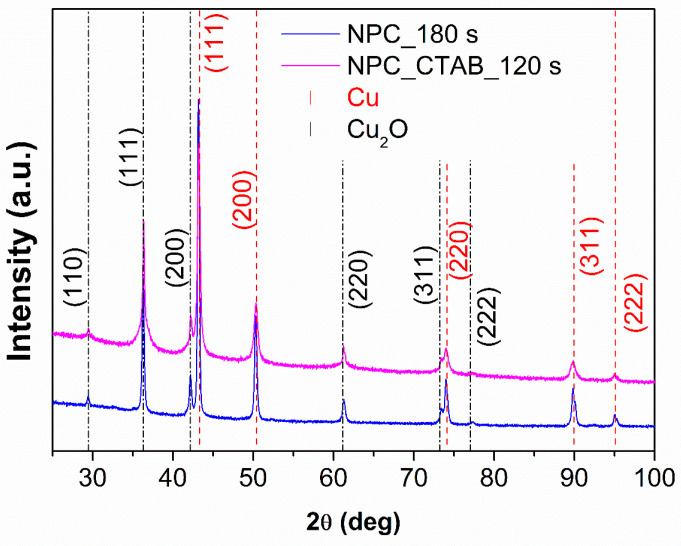
XRD patterns of NPC at 180 s without CTAB (blue line) and NPC at 120 s with CTAB (violet line).

The XRD patterns of NPC_180 s and NPC_CTAB_120 s are reported in Figure 4; the main intense peaks were attributed to the typical fcc structure of pure Cu, while the reflections with lower intensity were attributed to the fcc structure of Cu_2_O. It is worth mentioning that the formation of Cu_2_O results from the rapid surface air oxidation of the sample, which occurs within a few minutes of preparation. This is evident from the color change in the sample, which shifts from a coppery red to brown in a short period of time. Rietveld analyses were performed on these patterns to estimate the lattice parameter and the weight fraction of phases; the results are reported in Table 2.

The lattice parameters of the phases are consistent with those of pure Cu (i.e., 3.6150 Å) and Cu_2_O (i.e., 4.2670 Å) within the experimental error for both samples. The phase weight fractions show only a slight difference. The sample treated with CTAB exhibits a marginally higher oxide content, which can be attributed to the increased surface area, resulting in enhanced oxidation due to greater exposure to air.

### 3.3. SERS Analysis

**Figure 5:**
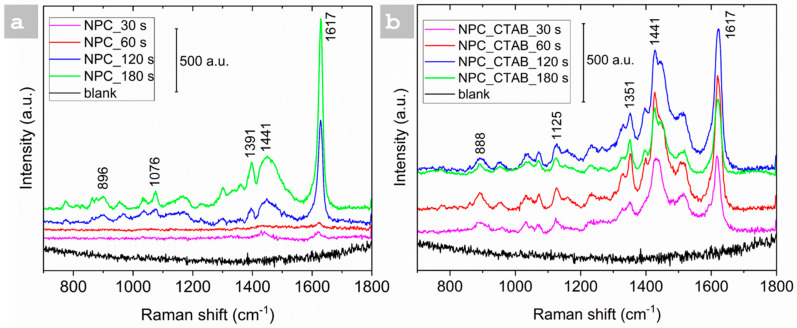
Effect of deposition time on SERS active NPC Substrate (**a**) NPCs without CTAB, (**b**) NPCs with CTAB.

The SERS activity of NPC substrates was evaluated by immersing them in a 10^−4^ M MB solution, serving as a probe molecule. The SERS spectra were acquired by directing the laser light onto the pore wall of the three-dimensional porous copper films. All the samples were initially tested with the same concentration of MB to identify the most active substrate, with and without the addition of CTAB, as illustrated in Figure 5. The NPCs deposited for 180 s without CTAB exhibited the most intense peak at 1628 cm^−1^ compared to the substrates prepared at 30, 60, and 120 s, as shown in Figure 5a. Clear Raman bands at about 449, 628, 1394, and 1617 cm^−1^ could be assigned to the C-N-C skeletal bending, C-C and C-N stretching, and C-N-S skeletal deformation of MB molecules, respectively [44]. Following this, the NPC substrate deposited for 180 s was selected to determine the low detection limit for MB. The same procedure was repeated for the NPC samples prepared with CTAB, and it was observed that the NPCs deposited at 120 s showed the most intense peak at 1617 cm^−1^, as depicted in Figure 5b, and they were selected for further investigations. Based on the morphological analysis, it has been observed that the dendrites of the NPC structures can cluster together, forming SERS hot spots. The presence of numerous slender dendrites in the porous copper, particularly when combined with CTAB, results in reduced spacing between them. This closer arrangement facilitates the generation of more surface plasmons, which enhances SERS activity. As a result, NPCs prepared with CTAB enhance SERS activity.

Furthermore, the SERS spectra clearly show unique peaks associated with MB molecules, indicating that the NPC films exhibit effective SERS active sites throughout all the samples. The morphological analysis illustrated in Figure 1a’–h’ indicates that extended deposition times enhance the formation of the porous structure. The notable SERS activity is primarily influenced by the optimal parameters of the NPC deposits and the plasmonic surfaces within the porous copper framework. Furthermore, the interfacial growth of the NPC film with CTAB at 180 s appears to hinder further deposition, resulting in a thinner film.

The reduction in thickness induces a modification of the sample morphology, which in turn affects the SERS activity of the thin films. Specifically, the pore–dendrite framework of the NPCs provides a high density of low-coordination sites, such as edges, steps, and kinks, that interact strongly with the target molecules [10,45]. This advantageous condition is more effectively realized at longer deposition times, leading to the formation of thicker thin films. Thereby, the peaks are less intense compared to 120 s, as illustrated in Figure 5b.

Moreover, the reproducibility of the NPC samples was confirmed by performing triplicate experiments, each using a freshly prepared solution and a new sample. Reproducibility tests focused particularly on the most active substrates, namely NPC_180 s and NPC_CTAB_120 s. Figures S6a and S7a in the Supplementary Materials present the mean ± standard error (SE) of signal intensities for the 10^−4^ M MB concentration, serving as a representative case. In Figures S6b and S7b, analysis of variance (ANOVA) indicates that the responses across the three independent trials, conducted with distinct pieces of NPC under identical conditions, are statistically consistent, with no significant differences observed in substrate performance.

**Figure 6:**
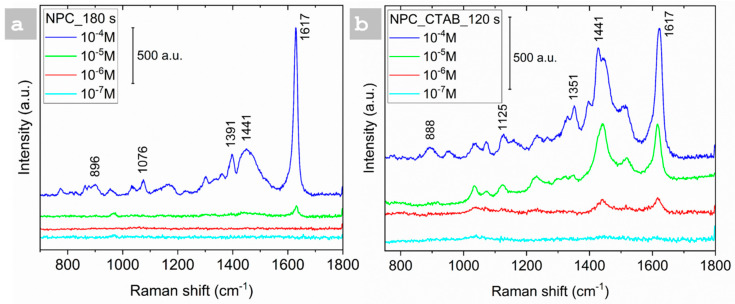
SERS sensitivity of MB using a concentration range of 10^−4^–10^−7^ using NPCs: (**a**) NPCs without CTAB and (**b**) NPCs with CTAB.

The Raman spectra of MB were evaluated within the concentration range of 10^−4^ to 10^−7^ mol/L to assess the SERS sensitivity of NPC film. Two different types of NPC-deposited films were used: one deposited at 180 s without CTAB and the other at 120 s with CTAB, as depicted in Figure 6. In Figure 6a, the spectra from the NPC-deposited film for 180 s without CTAB displayed a distinct peak that decreased in intensity as the MB concentration decreased from 10^−4^ to 10^−5^ M. However, no peaks were observed at lower concentrations (10^−6^ and 10^−7^ M), indicating a low limit of detection (LOD) of 10^−5^ M. In contrast, the samples containing CTAB exhibited a distinct peak at a lower concentration of 10^−6^ M, indicating a significant amplification of the Raman signal at this concentration by achieving a limit of detection of 10^−6^ M. This signifies that CTAB plays a crucial role in amplifying the Raman signals and facilitating noticeable signals at lower concentrations relative to the samples deposited without CTAB. The intensity of the Raman band at ~1628 cm^−1^, corresponding to the aromatic ring stretching of MB, was selected as the reference peak for EF and LOD analysis. Although the peak intensity at 10^−4^ M appears stronger in the DHBT sample (Figure 6a), the CTAB-modified substrate exhibits a detectable signal even at 10^−6^ M (Figure 6b), indicating a lower LOD. This demonstrates that CTAB facilitates improved signal sensitivity at lower analyte concentrations despite not producing the highest signal at saturation levels. Therefore, the CTAB effect is more evident in enhancing the detection limit rather than absolute intensity at high concentrations.

Additionally, a detailed comparison of the SERS active copper substrate prepared via different methods using the different analytes was performed in Table 3. Hu et al. [46] investigated the use of nanoporous copper (NPC) as a surface-enhanced Raman spectroscopy (SERS) substrate to enhance its enhancement factor (EF) for improved detection limits. In his study, Cu ion irradiation is applied to effectively increase the enhancement factor of NPC, resulting in a 4.4 × 10^4^ increase in the EF and a detection limit of 10^−4^ for rhodamine achieved by creating additional “hot spots” and roughening the NPC’s surface. Diao et al. [47] employed co-sputtering Cu/Ti followed by a dealloying process to synthesize nanoporous metals (NPMs) with tailored microstructures. The ideal NPC film demonstrated outstanding SERS performance for rhodamine 6G (R6G), with a low detection limit of 10^−9^ M and strong uniformity and repeatability. The enhancement factor was 4.71 × 10^7^, which is higher than Au substrates and equivalent to Ag systems.

In another study, Xu et al. [48] investigated the SERS activity of rhodamine 6G (R6G) Raman probes on gradient porous copper (Cu) substrates. Their study revealed an impressive SERS enhancement factor of 6.63 × 10^12^, along with a limit of detection of 2.10 × 10^−17^ mol/L, surpassing the performance of other porous SERS substrates.

Chen et al. [32] conducted a systematic investigation into the formation of nanoporous copper (NPC) through the selective corrosion of a single-phase Cu_30_Mn_70_ alloy in hydrochloric acid (HCl) aqueous solutions. The ability to tune nanopore sizes significantly enhanced the SERS effects of NPC. At the optimal nanopore size, the SERS enhancement factor achieved was approximately 1.85 × 10^5^, which is comparable to that of nanoporous gold with a limit of detection (LOD) of 10^−5^ mol/L for rhodamine and crystal violet. Li et al. [49] successfully prepared free-standing nanoporous copper foils (NPCFs) with a thickness of approximately 1.4 μm and an area of up to 10 cm^2^. Due to their nanoporosity, these NPCFs exhibited exceptional performance as SERS substrates, achieving an enhancement factor of 2.52 × 10^5^ while detecting rhodamine at a concentration of 10^−6^ M.

Yang et al. [50] employed a DHBT technique to fabricate dual-functional porous copper (Cu) films, enabling the in situ monitoring of electrocatalytic reactions through SERS. The resulting porous Cu films exhibited remarkable sensitivity, achieving a detection limit of 10^−6^ M for methylene blue while demonstrating excellent reproducibility. A detailed comparison of SERS active copper substrates is also reported in Table 3.

Although the LOD and EF values obtained in this work are lower than those reported in some earlier studies, this outcome can largely be attributed to differences in the fabrication methods used. Most of the reported high-performance Cu-based SERS substrates are produced via dealloying, a top-down approach that generates nanoscale porosity and fine structural features, which allow highly favorable plasmonic properties. In contrast, the samples presented here were synthesized through electrodeposition, a bottom-up method that typically leads to micrometer-scale pores and ligaments. While this results in inherently lower SERS activity, discussing these differences remains important in order to place the present results within the wider context of copper-based SERS research.

## 4. Conclusions

In this study, 3D NPC films were successfully fabricated using electrodeposition coupled with a DHBT approach, demonstrating their potential as effective SERS substrates for detecting the highly toxic dye methylene blue. The morphology of the NPC films was tailored in the presence of CTAB additives and adjusting the deposition time, both of which play crucial roles in the formation of bubble templates that induce the hierarchical structure. The SERS activity of the porous Cu films was significantly affected by the deposition time and the presence or absence of CTAB. The organic additive CTAB enhanced the formation of the NPC films, resulting in a thinner pore structure and finer dendrites approximately in the range of 12.8 to 24 µm, which contributed to their superior SERS activity at a deposition time of 120 s. In contrast, the nanoporous copper films prepared without CTAB demonstrated SERS performance at a longer deposition time of 180 s, resulting in a thicker dendritic structure that impacted their overall performance. Remarkably, the NPC films exhibited high sensitivity of methylene blue having LOD 10^−6^ mol/L with an enhancement factor of 2.5 × 10^3^, good reproducibility, and activity for SERS detection. Furthermore, the pore size and wall thickness of the copper films could be precisely tailored by varying both the electrodeposition time and the presence of the surfactant CTAB. The resulting NPC films with varied pore sizes and wall thicknesses offer a simple and fast way for producing NPC SERS substrates with adjustable morphology. The cost-effectiveness and facile fabrication process make the NPC-deposited film a promising candidate for various SERS applications.

## Figures and Tables

**Table 1:** Summary of NPC morphological analysis with and without CTAB.

Sample	Time of Deposition (s)	Pores Size (µm)	Dendrite Length (µm)
NPC_30 s	30	26.8 ± 9.4	2.9 ± 0.5
NPC_60 s	60	31.5 ± 12.7	4.3 ± 0.4
NPC_120 s	120	54.8 ± 17.2	18.4 ± 5.4
NPC_180 s	180	73.3 ± 32.2	28.2 ± 5.9
NPC_CTAB_30 s	30	12.8 ± 4.8	/
NPC_CTAB_60 s	60	23.5 ± 3.1	/
NPC_CTAB_120 s	120	24.4 ± 4.8	/
NPC_CTAB_180 s	180	less than 1.0	/

**Table 2:** Structural parameter and weight fraction of phases in NPC at 180 s without CTAB and NPC at 120 s with CTAB.

Sample	Lattice Parameter of Phases (Å)		Weight Fraction of Phases (wt.%)	
	Cu	Cu_2_O	Cu	Cu_2_O
NPC_180 s	3.62 ± 0.01	4.28 ± 0.01	74 ± 1	26 ± 1
NPC_CTAB_120 s	3.62 ± 0.01	4.24 ± 0.01	61 ± 1	39 ± 1

**Table 3:** A detailed comparison of SERS active copper substrates.

Substrate	Shape/Structure	Analyte	LS/PS/Diameter	LOD (mol/L)	EF	Ref.
Cu	butterfly wing scales	R6G	/	10^−8^	/	[33]
NP-Cu	Ribbon	R6G	~1 µm	10^−4^	4.4 × 10^4^	[46]
NPC	Film	R6G	41.6 ± 9.2 nm	10^−9^	4.7 × 10^7^	[47]
GP-Cu	Laminated	R6G	~24 nm	10^−17^	6.6 × 10^12^	[48]
NPCu	Ribbon	R6G/CV	~34 nm	10^−5^	1.85 × 10^5^	[32]
NPCFs	Foil	R6G	~200 nm	10^−6^	2.52 × 10^5^	[49]
PCu	Copper sheet	MB	43.1 µm	10^−9^	/	[50]
NPCs	Cu Disks	MB	12–73 µm	10^−6^	2.5 × 10^3^	This work

NPC: nanoporous copper; LS: ligament size; PS: pore size; LOD: limit of detection; EF: enhancement factor; GP-Cu: gradient porous copper; NPCFs: nanoporous copper films; MB: methylene blue; PCu: porous copper; R6G: rhodamine 6G; CV: crystal violet.

## Data Availability

Dataset available upon request from the authors. The raw data supporting the conclusions of this article will be made available by the authors upon request.

## Supplementary Materials

The following supporting information can be downloaded at https://www.mdpi.com/article/10.3390/nano15120945/s1, Figure S1. Bruker Alicona InfiniteFocus G6 in INRiM Nanometrology and Surface Metrology laboratory. Figure S2. Histogram of the height and thickness of the sample. Figure S3. Selection of “top” and “bottom” parameters. Figure S4. Evaluation of standard deviation σ. Figure S5. Three-dimensional topographies of samples without CTAB after deposition time of (a) 30 s, (b) 60 s, (c) 120 s, and (d) 180 s, and with CTAB after deposition time of (a’) 30 s, (b’) 60 s, (c’) 120 s, and (d’) 180 s. Table S1. Thickness evaluated by means of the histogram method. The high values of standard deviation are indicative of the inhomogeneity of the samples, which is more pronounced for the samples without CTAB. Figure S6. (a) The mean ± SE of intensities for batches of NPC_180 s at the concentration of 10^−4^ M MB; (b) the ANOVA (Analysis of the Variance) plot for the three batches. Figure S7. (a) The mean ± SE of intensities for batches of NPC_CTAB_120 s at the concentration of 10^−4^ M MB; (b) the ANOVA (Analysis of the Variance) plot for the three batches.
