# Supplementary material for: Nanoporous Copper Films via Dynamic Hydrogen Bubbling: A Promising SERS Substrate for Sensitive Detection of Methylene Blue
Source: Nanomaterials (Basel). 2025 Jun 18;15(12):945. doi: 10.3390/nano15120945 (PMC12196143; doi:10.3390/nano15120945)
Supplement: Supplementary file 1 [file nanomaterials-15-00945-s001.zip › nanomaterials-3655701-supplementary.pdf]

Supplementary Materials

Nanoporous Copper Films via Dynamic Hydrogen Bubbling: A Promising SERS Substrate for Sensitive Detection of Methylene Blue

Noor Tayyaba [1], Stefano Zago [1], Andrea Giura [2], Gianluca Fiore [1], Luigi Ribotta [2], Federico Scaglione [1,*] and Paola Rizzi [1]

[1] Dipartimento di Chimica e Centro Interdipartimentale NIS (Nanostructured Surfaces and Interfaces), Università di Torino, Via Pietro Giuria 7, 10125 Torino, Italy
[2] Applied Metrology and Engineering Division, Istituto Nazionale di Ricerca Metrologica (INRiM), Strada delle Cacce 91, 10135 Torino, Italy
* Correspondence: federico.scaglione@unito.it

Procedure details for thickness measurements on porous samples

Optical measurements of surfaces were performed in the Nanometrology and Surface Metrology laboratory at INRiM, the Italian National Metrology Institute. The instrument used was a Bruker Alicona InfiniteFocus G6 (Figure S1), using the Focus Variation measurement mode. This technology permits to reconstruct in a fast way the 3D surface of a multimaterial sample without contact and with high resolution. It combines the small depth of focus of an optical system with vertical scanning to provide topographical information from the variation of focus.

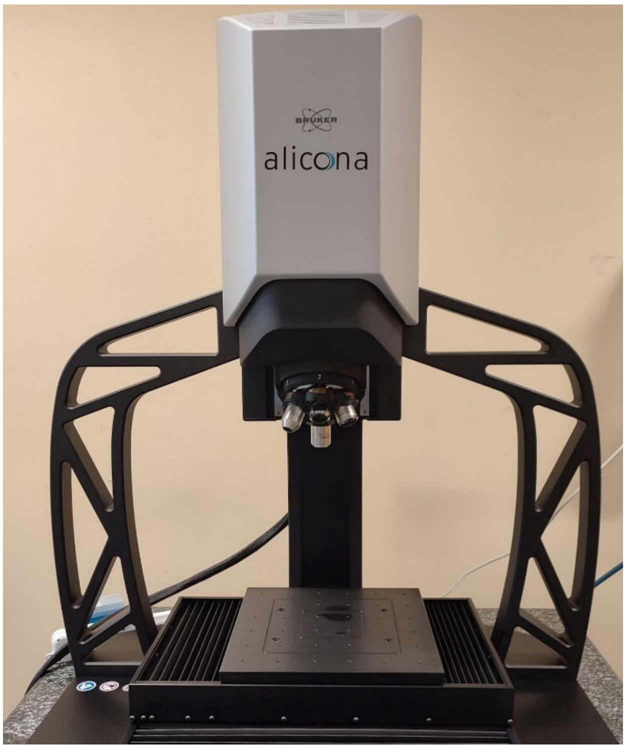

Figure S1. Bruker Alicona InfiniteFocus G6 in INRiM Nanometrology and Surface Metrology laboratory.

The topographies were performed by using a 4× objective with a working distance of 30 mm. All the measurements are the result of the stitching of 4×4 images, defining a

volume of about 14 mm × 14 mm × 0.5 mm in X, Y and Z directions, for a total of about 21 million points measured. The images were processed by using the metrological software Mountains Map v 10.0.

The thickness of the samples was evaluated by using the histogram method. This methodology allows to observe the density of the distribution of the data points in the surface being studied. The histograms built have 2000 bins, in which the highest peak refers to the height of the substrate, while the smallest peak is referred to as the top of the sample. In Figure S2 is depicted how the thickness of the sample is evaluated, by subtracting the quote of the most probable height of the sample to the substrate quote.

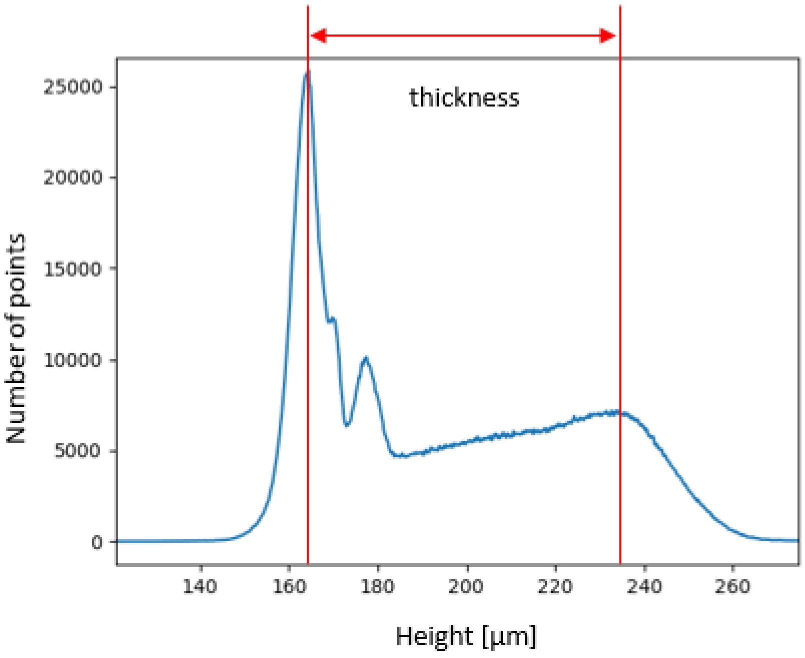

Figure S2. Histogram of height and thickness of the sample.

In order to evaluate the standard deviation of the histogram extracted by processing the topographies with Mountains Map, we develop a Python routine within the framework of the SurfILE project [35].

Figure S3 displays the elaboration parameters, which are:

- Bottom: lowest height value of the sample, considering only the features of interest.
- Top: most probable height value of the sample.

The data are then cropped to keep only the height values above the bottom, as reported in Figures S3 and S4.

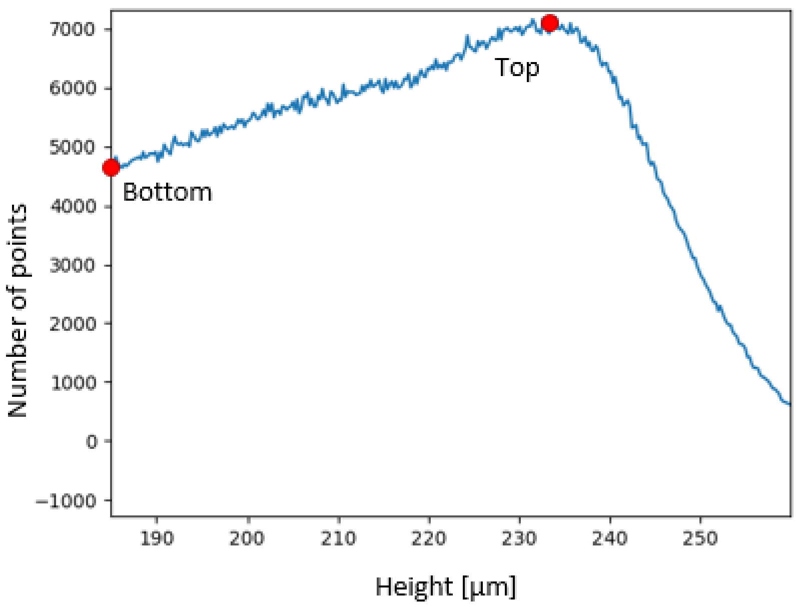

Figure S3. Selection of "top" and "bottom" parameters.

At this point, the distribution is integrated symmetrically with respect to the top until the 68% of the total points is reached (Figure S4). The choice of the percentage is arbitrary and corresponds to the confidence level; in this case approximating our distribution to a Gaussian one, it is useful for estimating a reasonable standard uncertainty.

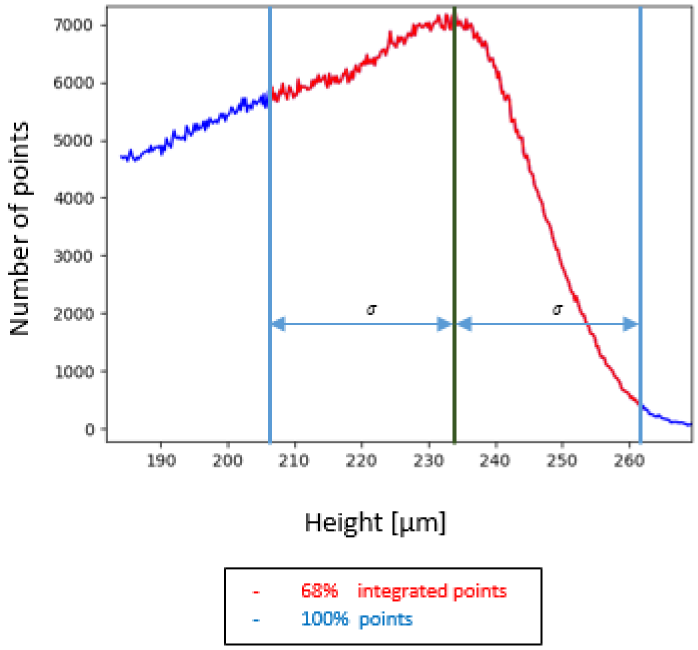

Figure S4. Evaluation of standard deviation σ.

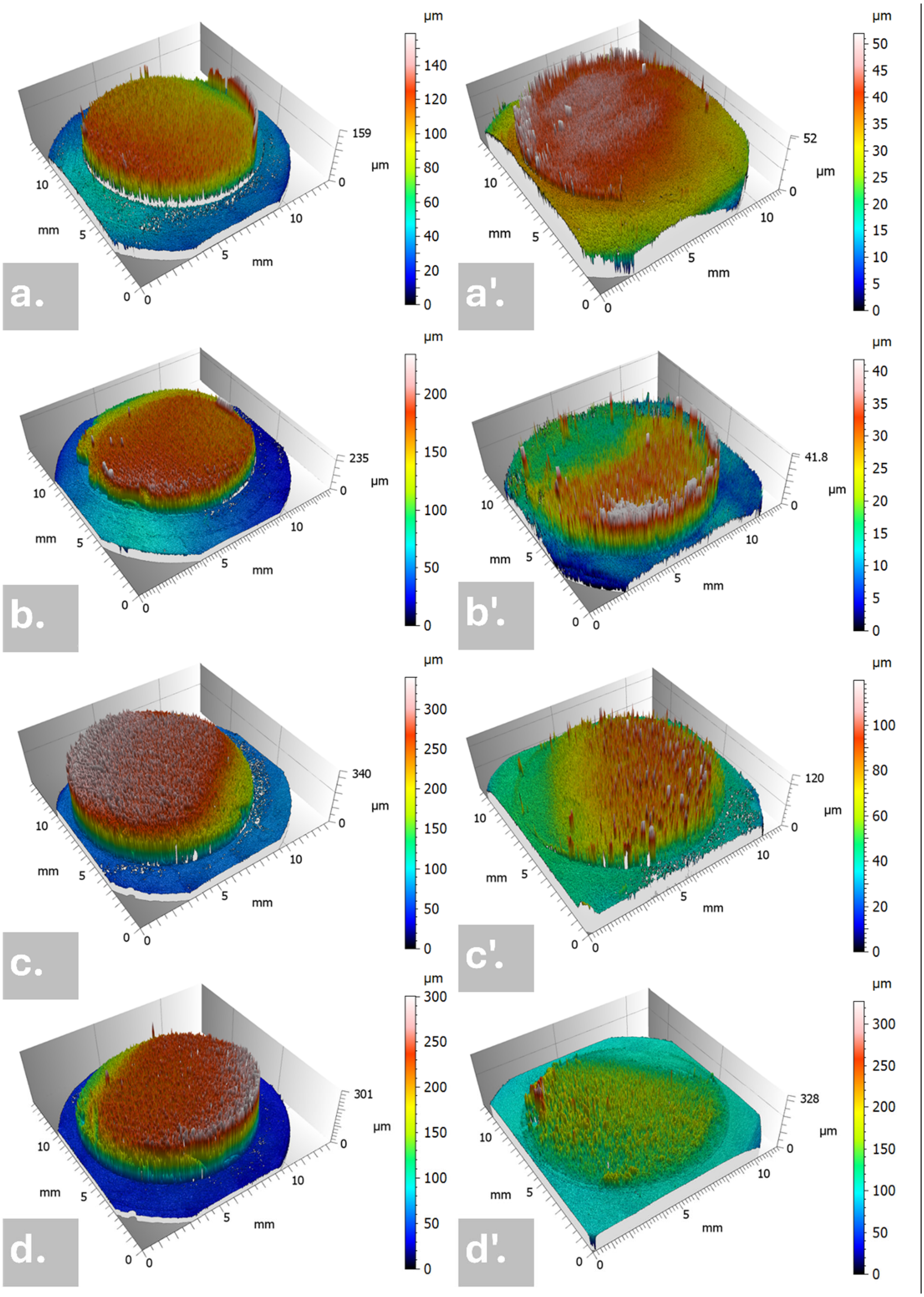

Figure S5. 3D topographies of samples without CTAB after deposition time of (**a**) 30 s (**b**) 60 s (**c**) 120 s and (**d**) 180 s, and with CTAB after deposition time of (**a'**) 30 s (**b'**) 60 s (**c'**) 120 s and (**d'**) 180 s.

Table S1. Thickness evaluated by means of histogram method. The high values of standard deviation are indicative of the inhomogeneity of the samples, which is more pronounced for the samples without CTAB.

Sample	time [s]	thickness [μm]	Standard dev. [μm]
NPC_CTAB_30s	30	3.28	6.50
NPC_CTAB_60s	60	6.26	4.78
NPC_CTAB_120s	120	29.4	9.54
NPC_CTAB_180s	180	43.6	18.4
NPC_30s	30	70.0	25.3
NPC_60s	60	130	30.9
NPC_120s	120	214	41.6
NPC_180s	180	185	41.4

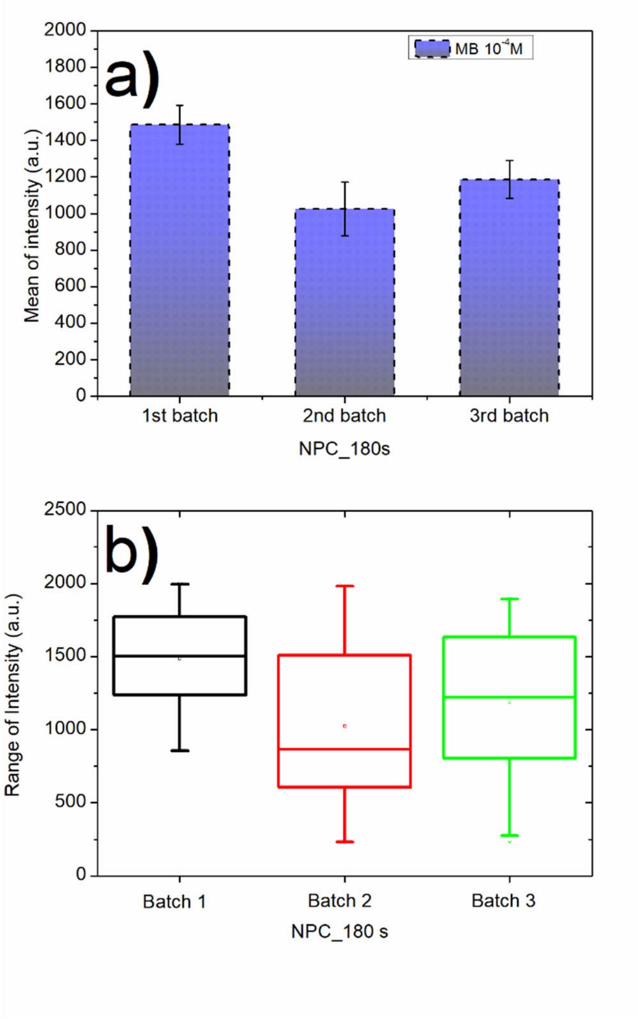

Figure S6. (**a**) The mean ± SE of intensities for batches of NPC_180 s at the concentration of 10^{-4} M MB; (**b**) the ANOVA (Analysis of the Variance) plot for the three batches.

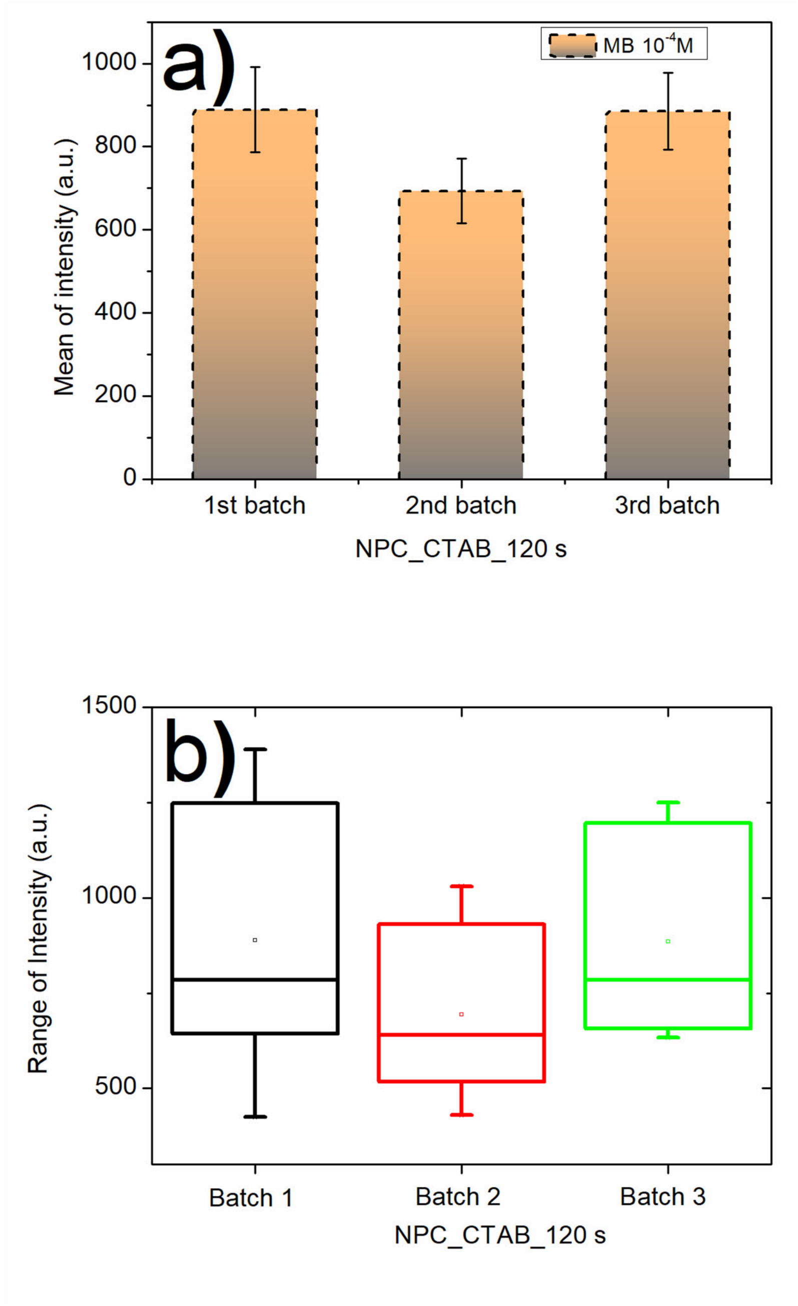

Figure S7. (**a**) The mean ± SE of intensities for batches of NPC_CTAB_120 s at the concentration of 10^{-4} M MB; (**b**) the ANOVA (Analysis of the Variance) plot for the three batches.
